# Field Flight Dynamics of Hummingbirds during Territory Encroachment and Defense

**DOI:** 10.1371/journal.pone.0125659

**Published:** 2015-06-03

**Authors:** Katherine M. Sholtis, Ryan M. Shelton, Tyson L. Hedrick

**Affiliations:** Department of Biology, University of North Carolina at Chapel Hill, Chapel Hill, North Carolina, United States of America; University of Utah, UNITED STATES

## Abstract

Hummingbirds are known to defend food resources such as nectar sources from encroachment by competitors (including conspecifics). These competitive intraspecific interactions provide an opportunity to quantify the biomechanics of hummingbird flight performance during ecologically relevant natural behavior. We recorded the three-dimensional flight trajectories of Ruby-throated Hummingbirds defending, being chased from and freely departing from a feeder. These trajectories allowed us to compare natural flight performance to earlier laboratory measurements of maximum flight speed, aerodynamic force generation and power estimates. During field observation, hummingbirds rarely approached the maximal flight speeds previously reported from wind tunnel tests and never did so during level flight. However, the accelerations and rates of change in kinetic and potential energy we recorded indicate that these hummingbirds likely operated near the maximum of their flight force and metabolic power capabilities during these competitive interactions. Furthermore, although birds departing from the feeder while chased did so faster than freely-departing birds, these speed gains were accomplished by modulating kinetic and potential energy gains (or losses) rather than increasing overall power output, essentially trading altitude for speed during their evasive maneuver. Finally, the trajectories of defending birds were directed toward the position of the encroaching bird rather than the feeder.

## Introduction

The flight capabilities of animals have long interested researchers, leading to an extensive literature on the aerodynamics and flight capabilities of birds, bats and insects examined in a diverse array of laboratory experiments. For example, wind tunnel experiments have measured flapping kinematics [1, 2], cost of transport and power requirements for flight [3–5] and quantified flow structures over the wing [6] and in the wake [7, 8]. Maneuvering course experiments [9–11] have revealed some of the turning capabilities of vertebrates engaged in low speed flight. In contrast, studies of animal flight capabilities in natural environments are far less common because of difficulties related to repeatability and of reliably obtaining precise, quantitative kinematic data at high temporal resolution in the field. Thus, although much is known about steady, level flight in controlled but highly artificial wind tunnel environments, it is less certain how these capabilities relate to natural flight behavior, especially at timescales in which turning, acceleration and deceleration occur and how they are used in day-to-day ecological contexts. However, continual advances in camera, inertial sensing and data-logging technologies now allow quantification of animal flight performance in the field at timescales of less than a tenth of a second, using high speed videography with multiple, calibrated cameras [12–14] or on-board inertial sensing and data recording[15] for larger animals able to easily lift the ~20 gram mass of current generation systems [16]. These new capabilities allow investigation into how animals deploy their flight capabilities during typical foraging, display and commuting behaviors.

Hummingbirds, whose small size and sustained ability to hover provide substantial physiological and biomechanical challenges that have been the subject of much laboratory investigation [17–20], are an excellent study system in which to examine how aerial maneuvering abilities and locomotor performance measured in the laboratory relate to field behaviors. We used high-speed videography to quantify the three-dimensional (3D) flight trajectories of free, wild hummingbirds around a stationary, outdoor feeder. This provided repeatable, quantitative observation of position as birds approached a feeder and departed from it either alone or while being pursued by another hummingbird. From recorded trajectories, we also computed velocity, acceleration, mass-specific kinetic and gravitational potential energy, and mass-specific kinematic (i.e. climb) power.

These data were then used to test the following hypotheses on how hummingbirds use their flight capabilities in different behaviors. First, we expected that birds departing from the feeder while being chased would exhibit greater accelerations, flight speeds and changes in kinetic and potential energy than birds departing alone. Producing larger flight forces associated with acceleration and higher speed is aerodynamically costly, and birds are only expected to meet these costs when necessary. Second, the measured mass-specific kinematic power output (i.e. rates of change in kinetic and potential energy) should be substantially below laboratory measurements of aerodynamic power requirements in hummingbirds since our field measurements do not include aerodynamic costs such as profile, parasite and induced power which can only be measured in lab. Furthermore, we do not expect birds to employ their maximal flight capabilities during typical behaviors such as those measured here, following the results of intraspecific contests in Cliff Swallows [13]. For similar reasons, the non-dimensional forces associated with acceleration in the field should be less than the maximum lift forces recorded from hummingbirds in laboratory tests. Finally, we hypothesize that the highest flight speeds will be found in the birds defending the feeder because they typically dive toward their target, using potential and muscle energy for locomotion, but that these will not exceed flight speeds recorded in hummingbird mating displays [12] where a maximal display of flight capabilities may be particularly beneficial and stored potential energy is also used.

In addition to biomechanical measures, our results also provide some information on the guidance and control used by the birds during these intraspecific interactions. Specifically, the pursuing bird defending the feeder might either aim for the food resource or for the encroaching bird. We hypothesize that the former is the preferred strategy because the food resource is stationary, simplifying the flight control and targeting requirements for the defending bird and might also be more readily defended by flying directly to it.

## Materials and Methods

### Ethics Statement

The hummingbird observation protocol was approved by the University of North Carolina at Chapel Hill Institutional Animal Care and Use Committee, no government permits were required for field observation of the species studied here.

### Hummingbird Recordings

**Fig 1:**
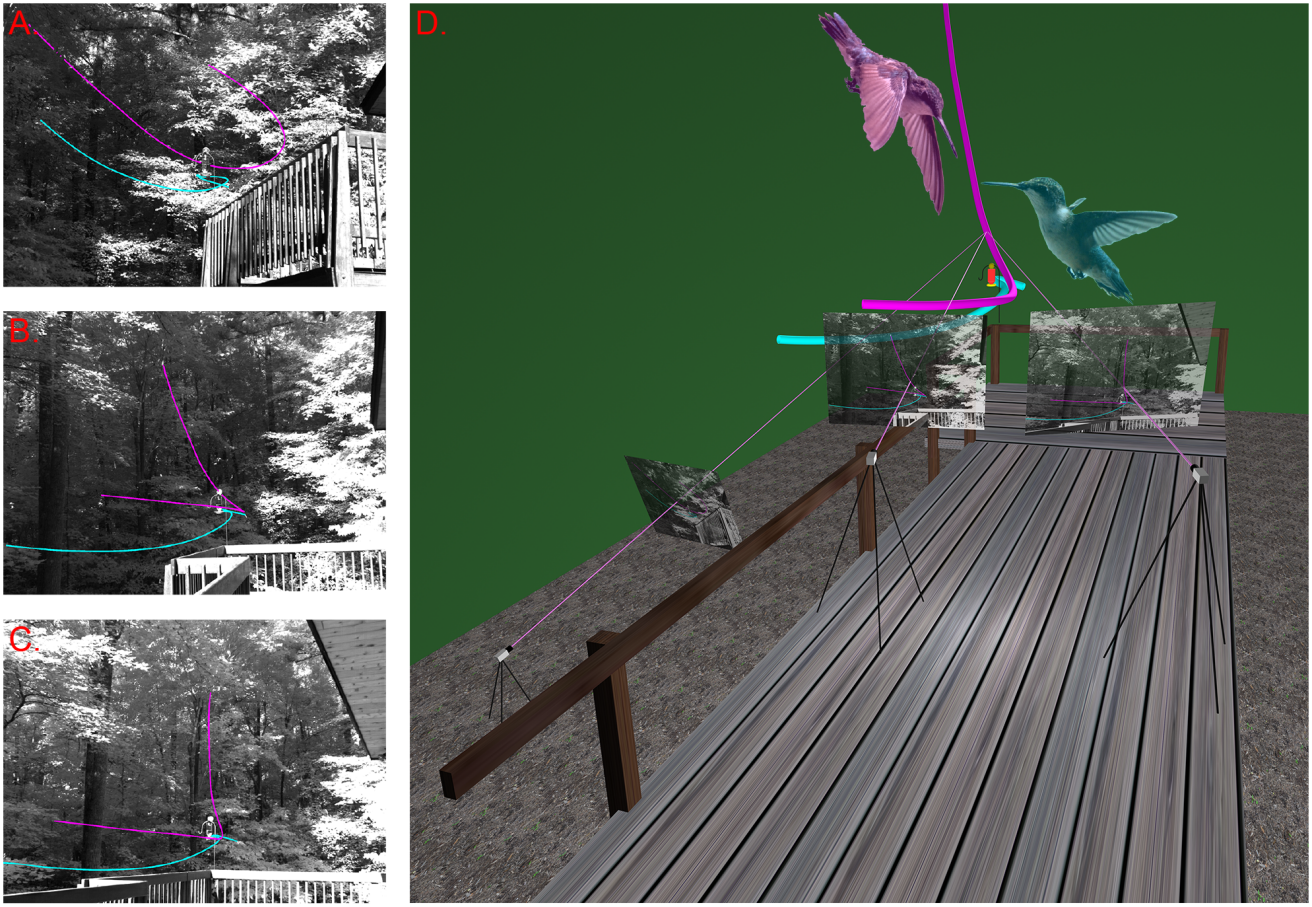
Video recording setup and example flight trajectories. Panels A-C show images from each of the three cameras in a recording setup; panel D shows a reconstruction of the 3D scene including the cameras, their positioning, their individual 2D images of the scene, the trajectory of the two hummingbirds in the 2D images as well as the 3D scene and triangulation rays identifying the 3D location of one bird at one instant from the 2D image information. The defending bird information is magenta while the chased bird is cyan; photographs (not to scale) of a defending and chased bird are included to show typical flight posture at the start of the interaction. Photo credit: Ellis Driver.

Using multiple camera high-speed videography, we recorded freely-behaving wild Ruby-throated Hummingbirds (*Archilochus colubris*, Linnaeus, 1758) during competitive interactions on private land in Orange County, North Carolina, USA (36° 0’ 29” N, 79° 5’ 51” W) with the permission of the landholder. The arena consisted of a wood deck with a single hummingbird feeder filled with clear 25% sugar water, located adjacent to a wooded area and bordered by a private residence (Fig 1). Video recording allowed sexing hummingbirds based on plumage in most cases. The hummingbird observation protocol was approved by the University of North Carolina at Chapel Hill Institutional Animal Care and Use Committee, no government permits were required.

We recorded on 14 separate mornings in the months of June, July, and August 2013 from approximately 8a.m. to noon at this location. The location of the cameras changed over the first two weeks to determine the best vantage points. Twenty-nine of the 51 videos analyzed had a camera configuration similar to that seen in Fig 1. Eighty-three separate videos were recorded, including both two-bird competitive interactions and single-bird arrivals and departures from the feeder. Thirty-three two-bird competitive interactions included the full two-bird interaction including pursuit by the defending bird and departure of the encroaching bird and were thus suitable for analysis. In these trials, we have designated the birds as either the encroaching, chased bird or the defending, chasing bird. Eighteen one-bird trials began with the bird hovering near the feeder then departing without provocation and were thus suitable for comparison with the chased bird from the two-bird interaction recordings. As previously reported for Ruby-throated Hummingbird competitive interactions [21], most defending and freely-departing birds were male while most chased birds were female. Respective counts of (male, female, undetermined) for the three behaviors were (31,2,0), (11,2,5), and (5,28,0). Note that the male-female distribution of defending and chased birds may reflect a behavioral case where the chase is the initial portion of a mating display rather than aggressive defense of a resource, though we did not observe any subsequent portions of the typical mating display sequence [22].

We used three synchronized high-speed cameras (N5r, Integrated Design Tools, Inc., Tallahassee, Florida, USA) recording 2336 x 1728 pixel images, generally at a 300 Hz frame rate for ~1.6 seconds. To provide longer observation durations, five trials were recorded at 200 Hz (~2.4 seconds of video) and one at 100 Hz (4.75 seconds of video). On these three cameras we used one 28mm and two 20mm Nikon lenses (AF NIKKOR 20 mm f/2.8D and AF NIKKOR 24 mm f/2.8 AIS, Nikon Inc., Melville, NY, USA). In order to collect 3D kinematic data, we used a structure-from-motion camera calibration routine [23]. Calibrations were obtained for each new setup at the start and end of each day's filming. Calibration sequences used a 1m wand moved within the field of view of all three cameras, with 20 hand-digitized wand points used in each calibration. The coordinate system z- axis was aligned to gravity using an additional recording of a rock thrown in the scene. The origin and the x- and y-axes were aligned to local environmental features ensuring a consistent reference frame for all trials.

To measure the wind, we placed a digital anemometer (HHF142, OMEGA Engineering, Inc, Stamford, Connecticut, USA) on the ground near the camera locations. To allow rotation about the vertical axis, the anemometer was mounted on a gimbal. A custom data logger recorded wind velocity at 1 Hz intervals as well as recording compass direction, time, and location. Only trials with a recorded wind speed of less than 1.5 m s^-1^ were analyzed; we did not include wind velocity in our kinematic analysis since it likely varied in magnitude and direction through the volume traversed by the birds.

### Video Analysis

After recording, the position of the birds in each video was digitized either by hand or using specially-written autotracker routines implemented using a combination of Python, OpenCV and MATLAB (The Mathworks, Natick, MA). Hand-digitizing via the MATLAB package DLTdv5 [24] was used initially and in cases where the autotracker was unable to recognize the birds for a short amount of time. Hand-digitizing was a slow task, averaging about two hours per 475 video frames. As the number of organisms, number of cameras, and frame rate increase, manual digitizing becomes a major challenge. Thus, the autotracker was developed to allow timely analysis of a larger number of recordings.

The autotracker functioned as follows: the scene background of each camera view was subtracted using a running average background subtractor configured to remove portions of the image that changed slowly (time scale longer than about 20 frames). Potential hummingbirds were then detected in each camera view using a Haar cascade [25] trained using data from the hand-digitized trials. Detections from all three cameras were then combined into specific 3D points along tracks by determining which pairwise and three-wise combinations of bounding box centroids produced three-dimensional positions with acceptably small stereo reconstruction errors (26 pixels rmse). This eliminated false detections such as those due to moving foliage and other sources of noise. Finally, all autodetected tracks were manually reviewed in order to remove obvious errors, and to merge tracks if the autotracker momentarily lost contact with the bird. The median rmse of all trials was 1.80 pixels, which corresponded to a median spatial 95% confidence interval volume of 5.75x10^-7^ m^3^. The effective recording volume was 347 m^3^, as measured by the minimum volume bounding box containing all hummingbird trajectory data points.

To remove digitization noise, the raw 3D position data were processed with a quintic smoothing spline implemented via the *spaps* function in MATLAB (The Mathworks, Natick, MA). For each data point, the error variance was extracted from the 3D reconstruction uncertainty and we iteratively increased the spline error tolerances, weighted by the error variances, to effect a low pass filter at 2.0, 3.0, and 4.0 Hz. Varying the low pass filter frequency did not affect any of our conclusions and produced only small variations in the data. Results presented here are from the 3.0 Hz low pass filter unless otherwise noted. We estimated derivatives of position with respect to time from the quintic spline polynomial. Velocity (***v***) and acceleration (***a***) were found by taking the first and second derivatives, respectively. We calculated the mass-specific kinetic (*KE*) and gravitational potential energy (*PE*) of the hummingbirds at any given time by:

$$\frac{KE}{m}=\frac{1}{2}{\left|v\right|}^{2}$$ (1)

$$\frac{PE}{m}=gh$$ (2)

where *m* is body mass, *g* is gravitational acceleration and *h* is elevation. We also computed mass-specific kinematic (*P*_*k*_) (i.e. climb) power as:

$$\frac{P_{k}}{m}=\frac{\Delta (\frac{1}{2}{\left|v\right|}^{2}+gh)\;}{\Delta t}$$ (3)

where *t* is time. In addition to our trajectories and mass-specific power estimates, two measures of maneuverability were computed: the angular velocity of the trajectory, i.e. the rate of change in direction of the trajectory and the centripetal force developed by the bird. Angular velocity was computed from instantaneous radius of curvature (*r*) directly from ***v*** and ***a*** by

$$r=\frac{{\left|v\right|}^{3}}{\sqrt{{\left|v\right|}^{2}\;{\left|a\right|}^{2}\;-\;{\left(v\;a^{'}\right)}^{2}}}$$ (4)

where ***a′*** is the transpose of ***a***. We then calculated angular velocity (*w*) by

$$w=\frac{\left|v\right|}{r}\;$$ (5)

Centripetal force (*F*) in body weights is then

$$F=\;\frac{{\left|v\right|}^{2}}{gr}.$$ (6)

and was adjusted to *F’*, the force produced by the bird only, by removing the gravitational contributions to *F* as described in [13]. Note that because angular velocity is undefined when flight speed is equal to zero, we only computed this quantity when the speed of the trajectory was greater than 1.0 m s^-1^.

### Trajectory analysis

We began analysis of the individual flight trajectories by first dividing them into three groups: trajectories from 1) freely-departing birds that approached the feeder and departed alone, 2) birds that approached the feeder and were chased away from it by a defending bird and 3) the defending birds. Next, members of each group were aligned to a common timebase with a start time determined by group-specific features. For the freely-departing and chased birds, timebase *t*_*c*_ was established by setting *t*_*c*_ = 0 to the instant at which the bird achieved a flight speed of 0.4 m s^-1^ during departure. Note that a flight speed of 0.0 m s^-1^ was not used because not all chased birds came to a complete stop. Two of the 33 chased birds never slowed to 0.4 m s^-1^ and these trajectories were not examined further.

**Fig 2:**
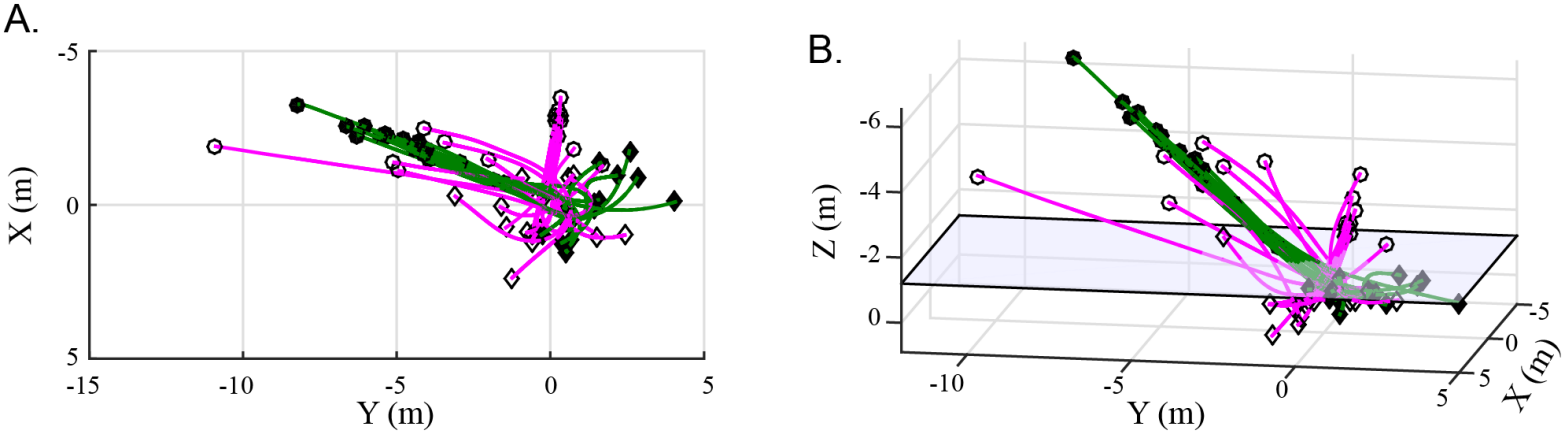
Defending bird trajectories and their classification. Circles indicate the starting position of the bird trajectory and diamonds its end position. Green lines with solid markers represent defenders using the perch, which was not within the 3D reconstruction volume. The pink lines with open markers represent other defender trajectories. A) A top-down view of the flight trajectories of the defending birds. B) A 3D view of the defending birds. The transparent blue plane at Z = 1.2m represents the approximate end of the uniform descent period of defender trajectories.

Defending birds typically entered the recording volume from above at a substantial speed. Timebase *t*_*d*_ was established for these trajectories by setting *t*_*d*_ = 0 to the instant at which the defending bird passed through a horizontal plane approximately 0.75 meters above the feeder and exactly 1.2 meters above the fixed rail where the feeder was mounted. This plane was chosen by examination of the set of defending bird trajectories, which revealed a period of constant slope descent which typically ended at this elevation (Fig 2). Once a common timebase was established for each group, average trajectories for each of the three groups were constructed by averaging the vertical and horizontal velocity and acceleration information of all the trials in the group for each.

Finally, we examined the question of whether the defending bird flies towards the chased bird or towards the feeder by computing the 3D angle between the trajectory of the defending bird and the direction of the feeder along with the 3D angle between the trajectory and the position of the chased bird. We used a one-sample t-test of the average difference between these 3D angles during the sustained descent phase (time less than 0) described above to assess the guidance decision of the defending bird.

### Statistical analysis

Specific statistical comparisons of trajectory values among the different flight groups were made by two-tailed, unpaired t-tests computed in MATLAB r2013b. Comparisons of defending and chased birds were by two-tailed, paired t-tests. Note that the identity of individual birds could not be established from the video data, so our comparisons are of bird trajectories rather than birds. A maximum of five birds were observed simultaneously in the vicinity of the feeder, so the trajectories were likely produced by five or more individual animals but fewer than the number of trajectories recorded, which was 18 to 33 depending on the behavior. The p values reported in the tables use the number of trajectories in determining the degrees of freedom. The effect of a reduction in degrees of freedom due to multiple recordings from the same bird can be estimated by assuming fewer degrees of freedom and examination of a table of t-distribution critical values; a reduction to only 5 degrees of freedom in both cases leads to roughly an order of magnitude increase in the p value.

Results in the text and tables are presented as mean ± standard deviation with a p value if a statistical comparison is called for. In contrast, trajectory results in figures are typically presented as the mean trajectory with error bars giving the standard error. This approach was taken because non-overlapping standard errors allow visual identification of cases likely to prove significantly different in a t-test whereas overlapping (or non-overlapping) standard deviations provide no such information.

## Results

### Trajectory classes

Freely-departing bird trajectories were recorded from birds leaving the feeder without being chased away. We acquired 18 videos of this class of trajectory; in 11 of these the hummingbird was male, in two it was female, and in five videos the sex of the bird could not be identified. These trajectories departed the feeder in a variety of directions, flying upward in 13 trials, flying level in three trials, and flying downward in two trials.

We acquired 33 videos of two-bird competitive interactions, which capture the flight trajectories of chased and defending birds. The videos start with the chased bird either approaching the feeder (12 of 33 trials), or hovering near it. The defending bird starts above the feeder and flies downward (in all but one trial) towards the chased bird while the chased bird is still approaching the feeder or while it is hovering nearby. The chased bird then departs from the feeder, flying downward in ten trials, flying level in 20 trials, and flying upward in three trials. For the chased bird trajectories, there were five recordings where the bird was a male and 28 videos where the bird was a female. For the defending bird trajectories, there were 31 videos where the bird was a male and two videos where the bird was a female.

We observed that the defending birds used a specific perch more often than any other location (16 out of 33 trials). This perch was located 6.5m above and 8.8m horizontal from the feeder, a total distance of 11.0m. In their initial approach to the feeder and chased bird, the defending birds descended towards the feeder at approximately the same rate. This period of uniform descent can be seen in Fig 2.

### Flight Speed and Velocity

**Fig 3:**
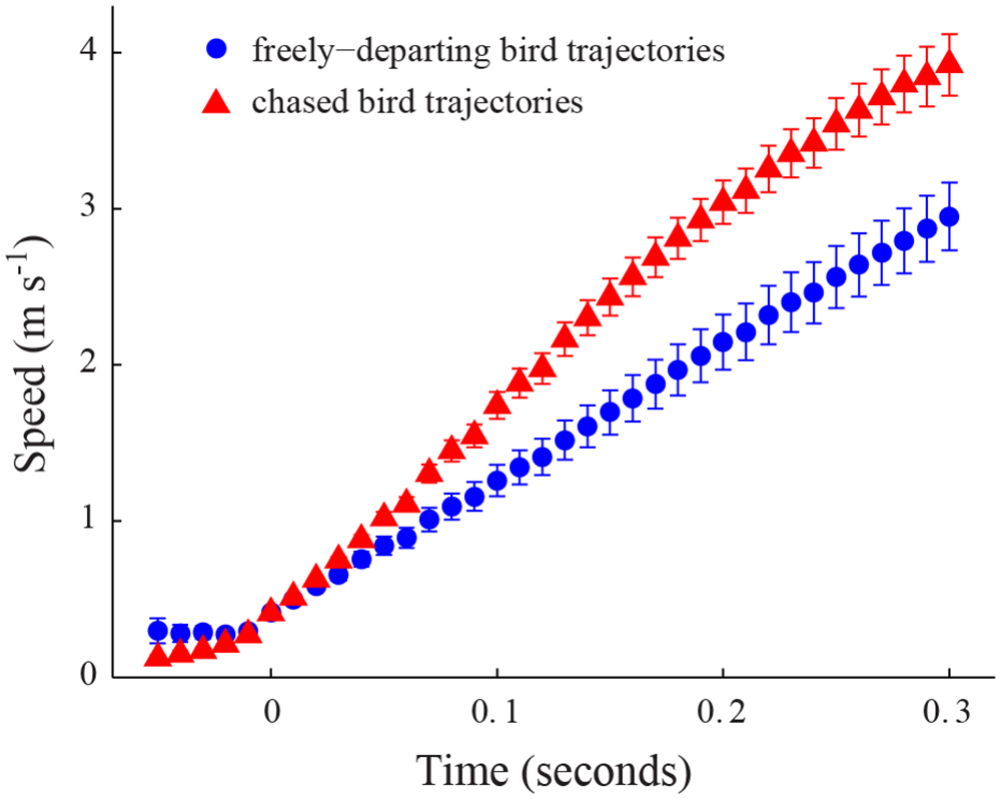
Flight speed in departure trajectories. The red triangle markers represent the mean flight speed of the chased bird after departing from the feeder. Error bars show the standard error (n = 31 trajectories at *t*_*c*_ = 0 and n = 30 at *t*_*c*_ = 0.29 seconds). The blue circle markers show the mean departure flight speed of freely-departing hummingbirds (n = 18 trajectories). Measurements of the flight speeds of both classes of birds were taken at different times after initial departure and a common timebase *t*_*c*_ created with 0 as the instant where the bird first reaches a speed of 0.4 m s^-1^.

**Fig 4:**
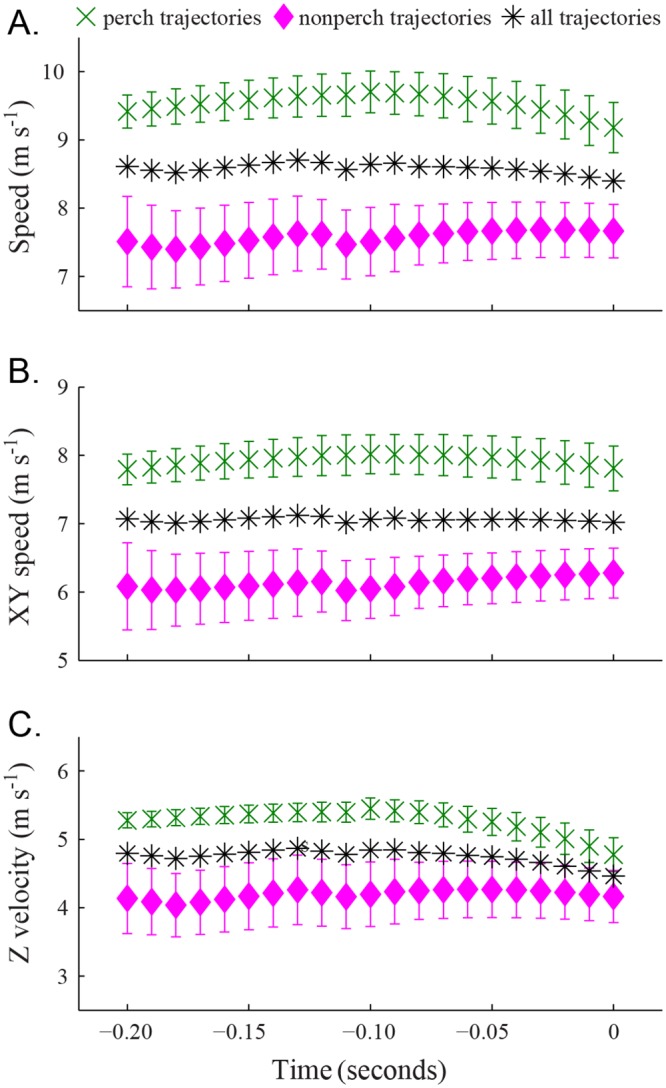
Flight speed in defending trajectories. The green cross markers show the mean flight speed of defending birds that approached the feeder from a frequently-used perch. Error bars show the standard error (n = 16 trajectories at *t*_*d*_ = 0 and n = 15 at *t*_*d*_ = 0.11 seconds). The pink diamond markers show the mean flight speed of all other defending trajectories (n = 17 at *t*_*d*_ = 0, n = 15 at *t*_*d*_ = 0.09 seconds, and n = 11 at *t*_*d*_ = 0.11). The black asterisk markers show the mean flight speed of all defending birds (n = 33 at *t*_*d*_ = 0, n = 30 at *t*_*d*_ = 0.09, and n = 26 at *t*_*d*_ = 0.20 seconds). (A) Gives the overall flight speed while (B) shows speed in the horizontal (XY) projection of the trajectories and (C) shows vertical velocity. Note that a positive vertical velocity is downward toward the ground and feeder. The trajectories were combined using a common *t*_*d*_ where 0 is the time at which the trajectory passed through Z = -1.2 m.

The feeder departure trajectories from chased birds showed a greater flight speed than those recorded from freely-departing birds (Fig 3, Table 1). For example, at 0.3 seconds after departure, the mean speed of all chased bird trajectories was 3.9 ± 1.1 m s^-1^ (mean ± standard deviation here and elsewhere) while the mean speed of all freely-departing trajectories was 3.0 ± 0.8 m s^-1^ (t-test, p = 0.0039). We found that defending trajectories originating at the typical perch (see above) differed from those originating at other locations with respect to their velocity profile (Fig 4, Table 2). Perch trajectories had greater overall flight speeds, with greater horizontal and vertical velocities. For all defending trajectory velocity comparisons, the differences in velocity between the two classes of defending trajectory lessened as the birds approached the feeder (Fig 4). Finally, maximum speeds recorded from defending bird trajectories were greater than those recorded in the matching chased bird trajectory (Table 3).

**Table 1:** Departing bird trajectories.

	Freely—departing bird trajectories (n = 18)	Chased bird trajectories (n = 31)	Statistical comparison (unpaired t-test)
Flight speed at *t*_*c*_ = 0.3s	3.0 ± 0.8 (mean ± s.d.)	3.9 ± 1.1	p = 0.0039
Maximum flight speed (m s^-1^)	5.4 ± 1.4	6.2 ± 1.3	p = 0.0629
*t*_*ma*_: *t*_*c*_ at maximum average acceleration, *t*_*c*_ ≤ 0.3s (s)	0.17	0.08	-
Acceleration magnitude at *t*_*ma*_ (m s^-2^)	9.1 ± 4.8	14.6 ± 5.7	p = 0.0025
Overall maximum acceleration (m s^-2^)	19.6	42.3	-
Kinetic + potential energy at *t*_*c*_ = 0.3s (J kg^-1^)	35.1 ± 2.1	35.2 ± 3.0	p = 0.6812
Kinetic energy at *t*_*c*_ = 0.3s (J kg^-1^)	4.7 ± 2.6	8.3 ± 4.2	p = 0.0046
Potential energy at *t*_*c*_ = 0.3s (J kg^-1^)	30.4 ± 1.1	27.2 ± 2.3	p < 0.0001
Mean kinematic power at 0 ≤ *t*_*c*_ ≤ 0.3s (W kg^-1^)	18.6 ± 6.8	20.3 ± 8.4	p = 0.5050
Maximum mean kinematic power 0 ≤ *t*_*c*_ ≤ 0.3s (W kg^-1^)	33.0	39.4	-
Maximum angular velocity (degrees s^-1^)	258.8 ± 107.4	334.8 ± 149.2	p = 0.0627
Flight speed at maximum angular velocity (m s^-1^)	1.5 ± 0.7	2.2 ± 1.4	p = 0.0556
Maximum gravity-adjusted centripetal force (body weights)	0.9 ± 0.3	1.6 ± 0.7	p = 0.0002
Flight speed at maximum gravity-adjusted centripetal force (m s^-1^)	3.1 ± 1.7	4.2 ± 1.5	p = 0.0242

**Table 2:** Defending bird trajectories.

	Trajectories beginning at the typical perch (n = 16)	Other trajectories (n = 17)	Statistical comparison (unpaired t-test)
Flight speed at *t*_*d*_ = 0 (m s^-1^)	9.2 ± 1.5	7.7 ± 1.6	p = 0.0085
Horizontal flight speed at *t*_*d*_ = 0 (m s^-1^)	7.8 ± 1.3	6.3 ± 1.5	p = 0.0040
Vertical flight speed at *t*_*d*_ = 0 (m s^-1^)	4.8 ± 1.0	4.2 ± 1.6	p = 0.1920
Mean of maximum flight speeds (m s^-1^)	10.1 ± 0.7	8.2 ± 1.7	p = 0.0003
Overall maximum flight speed (m s^-1^)	12.0	10.7	-
Mean acceleration at *t*_*d*_ = -0.2 (m s^-2^)	3.8 ± 3.8	3.5 ± 2.9	p = 0.8110
Mean acceleration at *t*_*d*_ = -0.1 (m s^-2^)	-1.0 ± 8.1	4.2 ± 4.3	p = 0.0350
Mean of maximum accelerations (m s^-2^)	10.1 ± 6.8	10.4 ± 3.9	p = 0.8898
Overall maximum acceleration (m s^-2^)	30.1	17.7	-
Mean of maximum decelerations (m s^-2^)	-19.5 ± 9.1	-15.1 ± 7.6	p = 0.1371
Overall of maximum deceleration (m s^-2^)	-28.4	-26.6	-
Kinetic + potential energy at *t*_*d*_ = -0.2s (J kg^-1^)	96.4 ± 8.6	80.5 ± 18.5	p = 0.0074
Kinetic energy at *t*_*d*_ = -0.2s (J kg^-1^)	44.7 ± 7.6	30.4 ± 16.3	p = 0.0061
Mean kinematic power at -0.2 ≤ *t*_*d*_ ≤ 0s (W kg^-1^)	-72.1 ± 53.8	-26.9 ± 47.7	p = 0.0156
Maximum mean kinematic power -0.2 ≤ *t*_*d*_ ≤ 0s (W kg^-1^)	-10.9	29.9	-
Maximum angular velocity (degrees s^-1^)	342.2 ± 161.1	304.1 ± 124.2	p = 0.4510
Flight speed at maximum angular velocity (m s^-1^)	3.7 ± 2.7	4.0 ± 2.5	p = 0.7674
Maximum gravity-adjusted centripetal force (body weights)	2.2 ± 0.5	2.1 ± 0.9	p = 0.7173
Flight speed at maximum gravity-adjusted centripetal force (m s^-1^)	6.9 ± 2.4	5.9 ± 2.1	p = 0.2944

**Table 3:** Defending *vs*. Chased bird trajectories.

	Defending bird trajectories (n = 31)	Chased bird trajectories (n = 31)	Statistical comparison (paired t-test)
Mean of maximum flight speeds (m s^-1^)	9.2 ± 1.6	6.3 ± 1.3	p < 0.0001
Maximum angular velocity (degrees s^-1^)	322.6 ± 142.4	334.8 ± 149.2	p = 0.7383
Flight speed at maximum angular velocity (m s^-1^)	3.9 ± 2.6	2.2 ± 1.4	p = 0.0006
Maximum gravity-adjusted centripetal force (body weights)	2.1 ± 0.7	1.6 ± 0.7	p = 0.0051
Flight speed at maximum gravity-adjusted centripetal force (m s^-1^)	6.2 ± 2.3	4.2 ± 1.5	p < 0.0001

### Acceleration

**Fig 5:**
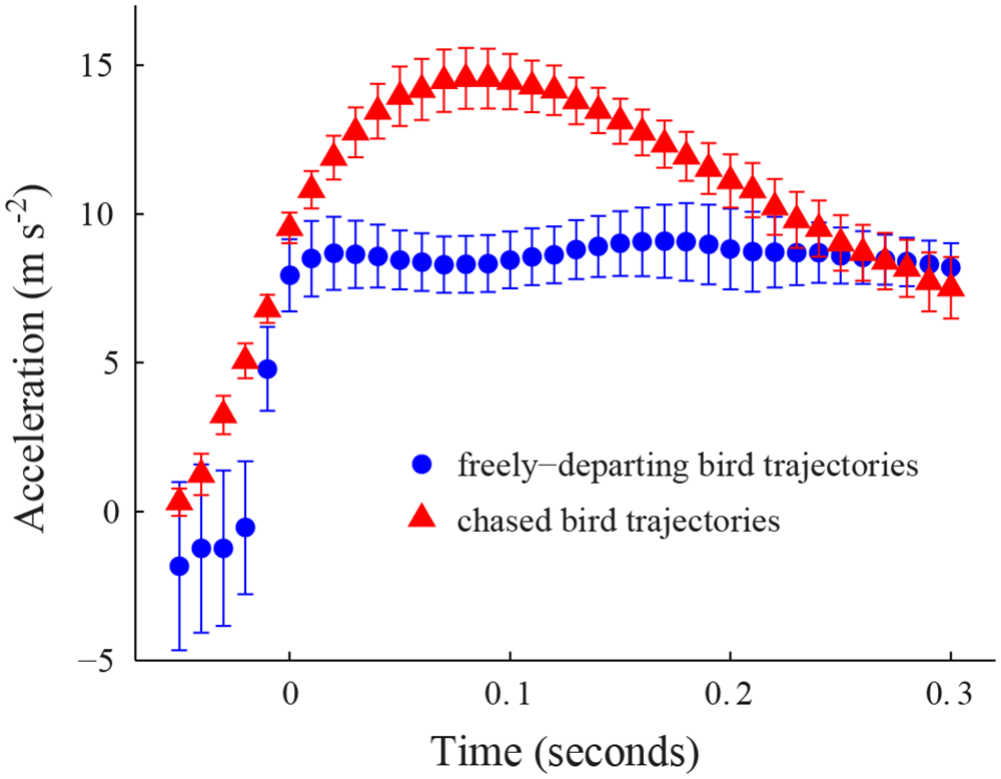
Acceleration in departing trajectories. The red triangle markers show the mean acceleration of the chased bird after departing the feeder (n = 31 trajectories at *t*_*c*_ = 0 and n = 30 at 0.29 seconds). The blue circle markers represent the mean acceleration after departure for freely-departing bird trajectories (n = 15). Error bars show the standard error at each time instant. Measurements of the flight speeds of both classes of birds were taken at different times after initial departure and a common timebase *t*_*c*_ created with 0 as the instant where the bird first reaches a speed of 0.4 m s^-1^.

We observed differences in acceleration between departures of chased bird trajectories and freely-departing trajectories (Fig 5, Table 1). The chased trajectories exhibited a higher initial acceleration during the first 0.25 seconds after departure, after which the two classes of departing birds had similar acceleration profiles. The initial accelerations were typically the largest magnitude events in the trajectory, thus the chased bird trajectories had significantly higher overall maximum accelerations compared to the freely-departing ones (t-test, p = 0.0025). The overall maximum instantaneous acceleration found within a chased bird trajectory was 42.3 m s^-2^ compared to 19.6 m s^-2^ for a freely-departing trajectory.

**Fig 6:**
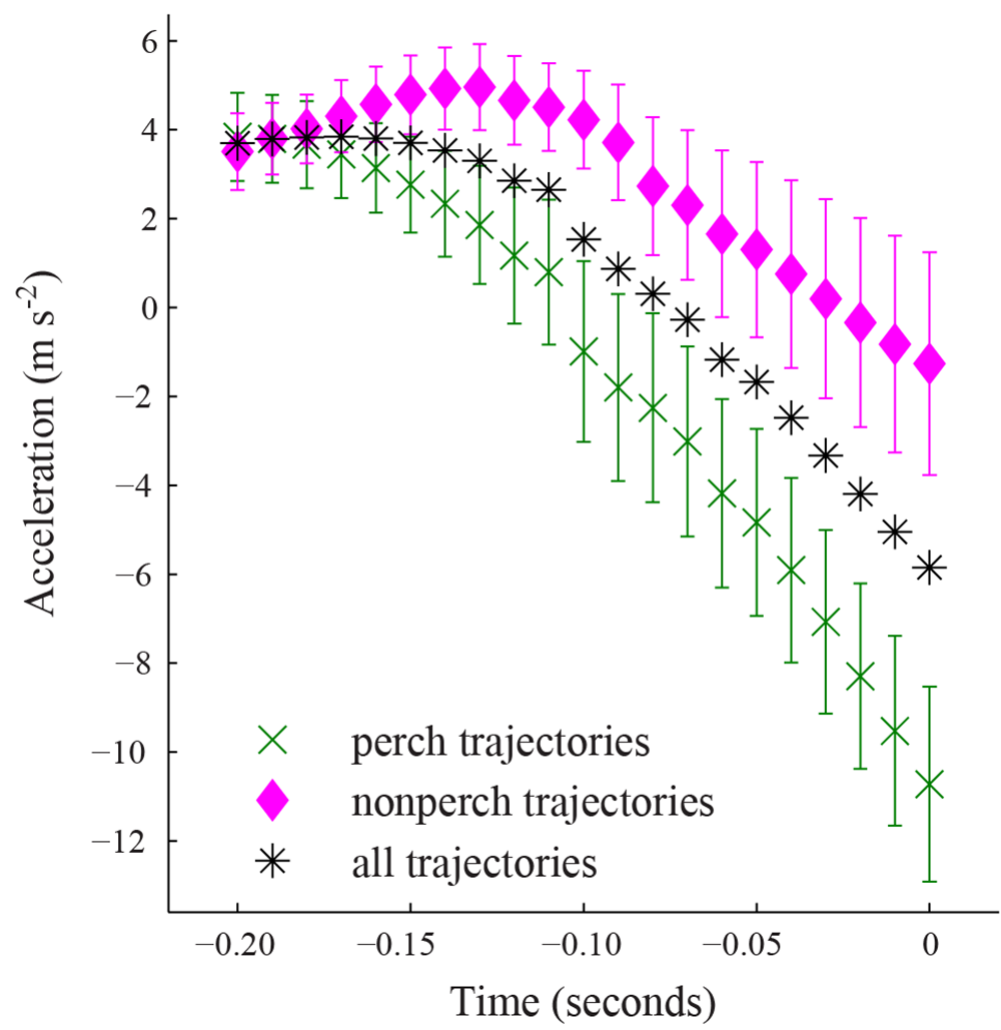
Acceleration in defending trajectories. The green cross markers show the mean acceleration of defending bird trajectories that approached the feeder from a specific perch (n = 16 trajectories at *t*_*d*_ = 0 and n = 15 at *t*_*d*_ = 0.11 seconds) while pink diamond markers show mean acceleration of all other defending bird trajectories (n = 17 at *t*_*d*_ = 0, n = 15 at 0.09 seconds, and n = 11 at 0.20 seconds). The black asterisk markers are the mean acceleration of all defending trajectories (n = 32 at *t*_*d*_ = 0, n = 30 at 0.09 seconds, and n = 26 at 0.20 seconds). Error bars show the standard error about the mean for the perch and nonperch data only.

The two classes of defending bird trajectories, perch and non-perch, produced similar magnitude accelerations when approaching the feeder, but perch trajectories began decelerating earlier (Fig 6, Table 2). The maximum accelerations for non-perch and perch defending birds were similar (Table 2) as were the deceleration magnitudes. In general, for the portion of the defending bird trajectories recorded in this study, the deceleration magnitudes are greater than the acceleration magnitudes.

### Changes in mass-specific energy state

**Fig 7:**
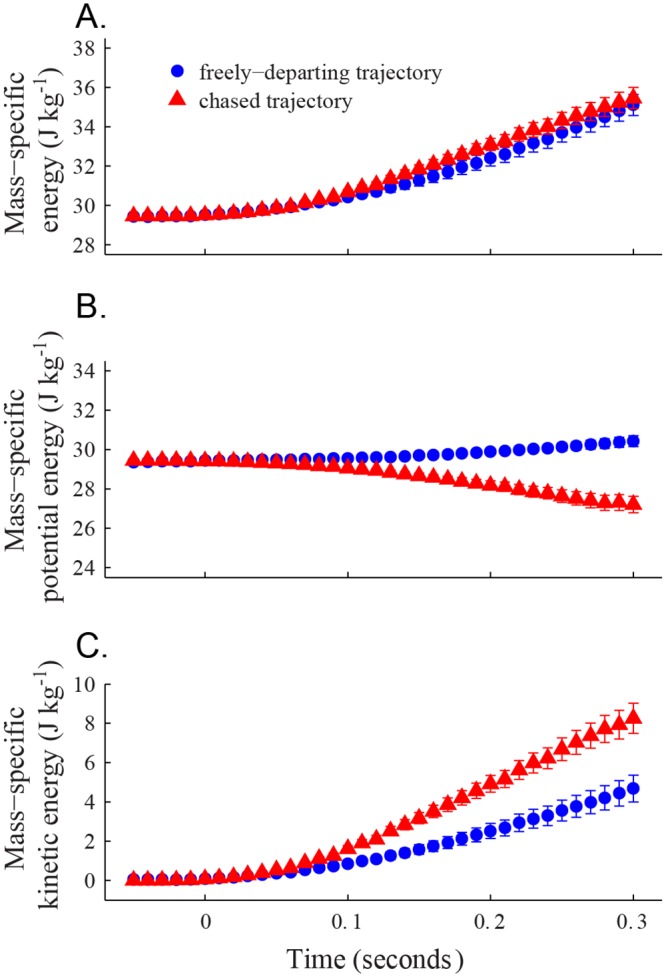
Kinetic and potential energy in departure trajectories. Here we show the (A) mass-specific energy, (B) potential energy, and (C) kinetic energy of hummingbird trajectories departing from the feeder location under different conditions. The red triangle markers show the chased bird after departing the feeder (n = 31 trajectories at *t*_*c*_ = 0 and n = 30 at 0.29 seconds). The blue circle markers show trajectories from freely-departing birds (n = 15). The starting positions of all departing trajectories were set to the same value (3m above the ground) to provide a common point of comparison for changes in energy.

Despite previously noted differences in acceleration profile, when comparing energy usage during feeder departures from chased bird trajectories versus those from freely-departing cases, we found that the two classes had similar overall mass-specific energy values (Fig 7, Table 1) although these were partitioned differently among potential and kinetic energy. Thus, at 0.3 seconds after departure, the mass-specific energies of the two classes of departing bird trajectory were not statistically different (t-test, p = 0.6812), but the mass-specific potential energies were significantly different (t-test, p<0.0001), as were the mass-specific kinetic energies (t-test, p = 0.0046). The chased trajectories tended to trade potential energy for kinetic energy compared to freely-departing cases by flying level or downward when departing from the feeder. The freely-departing trajectories tended to fly upward, resulting in a potential energy gain but lower flight speeds compared to chased bird trajectories.

**Fig 8:**
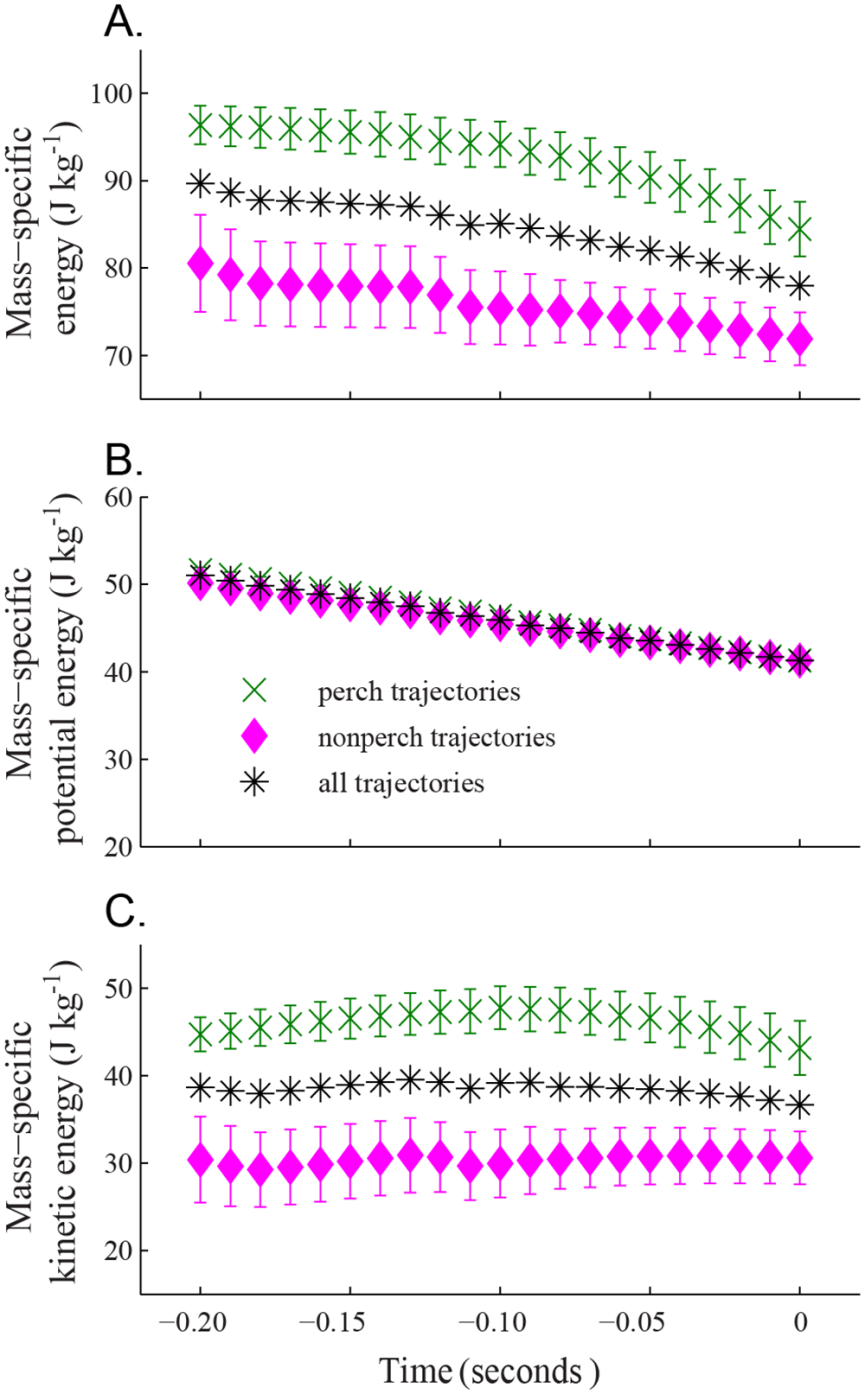
Kinetic and potential energy in defending trajectories. Here we show the (A) mass-specific energy, (B) potential energy, and (C) kinetic energy of trajectories from hummingbirds defending the feeder. The green cross markers show data from defending birds that approached the feeder from a specific perch (n = 16 trajectories at *t*_*d*_ = 0 and n = 15 at ≤ -0.11 seconds). The pink diamond markers show all other defending trajectories (n = 17 at *t*_*d*_ = 0, n = 15 at -0.09 to -0.20 seconds, and n = 11 at -0.20 seconds). The black asterisk marker represents all defending trajectories (n = 31 at time zero, n = 30 at -0.09 to -0.20 seconds, and n = 26 at -0.20 seconds). Error bars show the standard error about the mean for perch and nonperch trajectories. Unlike our treatment of departing bird potential energy, in (B) the starting positions of the birds were not set to a common identical initial value but are nevertheless quite similar.

We found that the two classes of defending bird trajectories, perch and non-perch, had similar mass-specific potential energy values (by definition in our timebase alignment), but had different mass-specific energy and kinetic energy values (Fig 8, Table 2). While approaching the feeder, both classes of defending trajectory decreased in elevation at similar rates, which resulted in potential energy decreasing uniformly upon descent. The perch trajectories were at higher flight speeds and thus higher kinetic energies.

We also measured the kinematic power output of the trajectories (i.e. the mass-specific rate of change in kinetic and potential energy). Chased and freely-departing trajectories had similar kinematic power outputs over the 0.3 second duration after initial departure (t-test, p = 0.5050; Table 1). The mean power output for chased birds was 20.3 ± 8.4 W kg^-1^, and the mean power output found for non-chased birds was 18.6 ± 6.8 W kg^-1^. The respective maximum kinematic power output found for the two classes was 39.4 W kg^-1^ and 33.0 W kg^-1^.

Mean kinematic power output from the defending bird trajectories was found for the 0.2 second range prior to the end of uniform descent. Unlike the departing birds, the defending birds’ kinematic power was typically negative during this time frame, usually by decreasing both speed and elevation. There were significant differences between the power outputs of perch and non-perch defending bird trajectory classes (t-test, p = 0.0156; Table 2). The mean kinematic power output for perch defender trajectories was -72.1 ± 53.8 W kg^-1^, compared to -26.9 ± 47.7 W kg^-1^ for other defender trajectories.

### Angular dynamics

**Fig 9:**
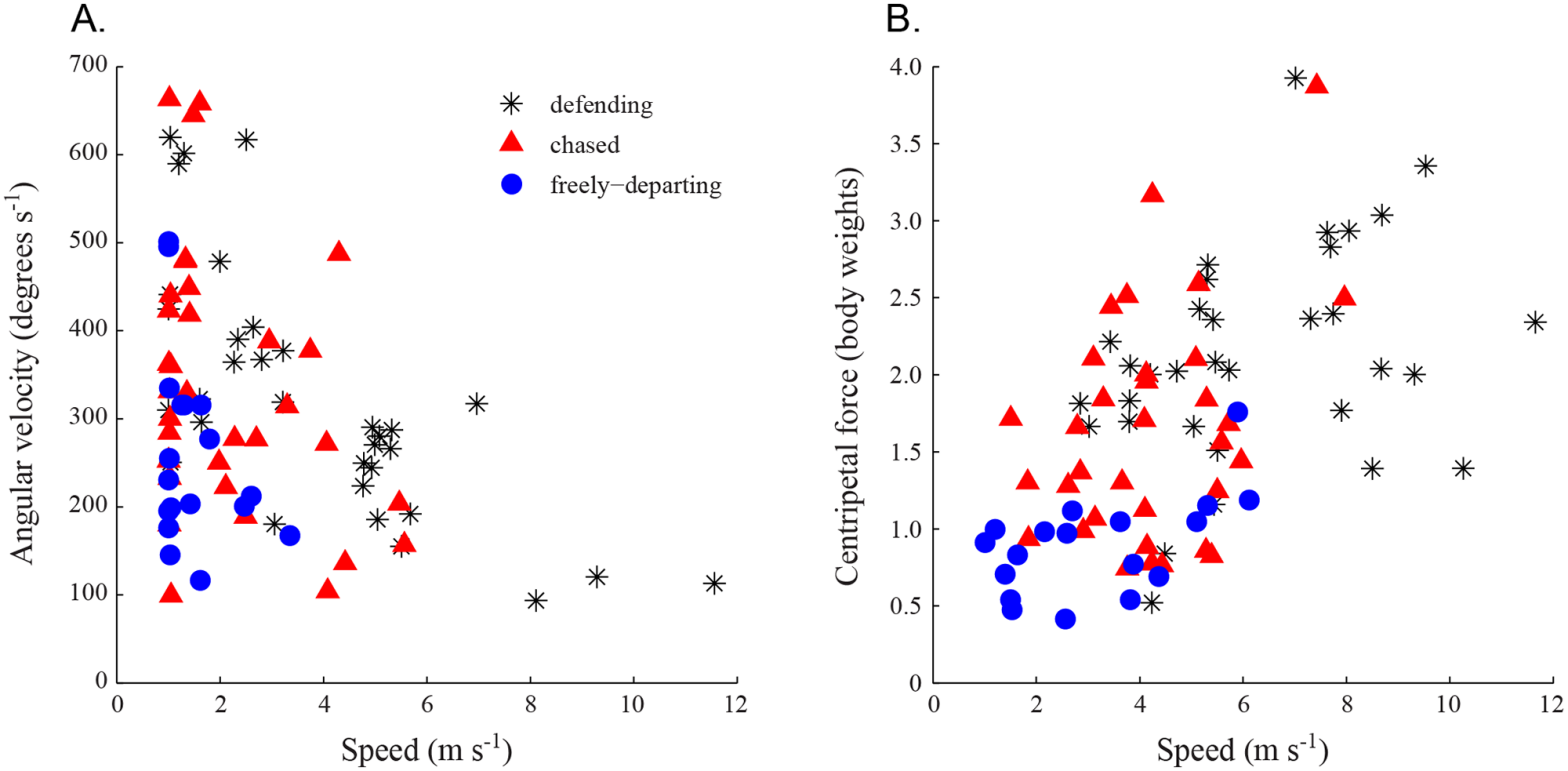
Turning rates and forces from all trajectories. Panel (A) shows the maximum rate of change in heading (i.e. angular velocity) and (B) the maximum centripetal force versus flight speed from the trajectories of hummingbirds defending the feeder and from those departing the feeder. The black asterisks are the defending birds (n = 33), red triangles are the departures of chased birds (n = 33) and the blue circles are freely-departing birds (n = 18). In both cases we identified the time in the trajectory where the maximum rate of change in flight direction (A) or maximum centripetal force (B) occurred and plotted the data against the speed at that instant. Computations in both cases were also limited to data points with speed > 1 m s^-1^.

Higher angular velocities in all trajectories tended to occur at lower speeds (Fig 9A). The maximum angular velocities of freely-departing and chased bird trajectories did not differ significantly (t-test, p = 0.0627; Table 1), and neither did the flight speeds at which these maneuvers occurred. However, differences appeared when these results were examined in terms of centripetal force rather than angular velocity (Fig 9B, Table 1). The maximum centripetal forces from freely-departing and chased trajectories differed significantly (t-test, p = 0.0002; Table 1) as did the speeds at which the forces were produced. Defending birds were similar to chased birds in their maximum angular velocities (Table 3), but differed in other measures of turning performance. The largest average centripetal forces were observed in defending bird trajectories and the overall maximum centripetal force (3.9 body weights) was a defending bird turning at a flight speed of 7.0 m s^-1^.

### Guidance and targeting

During the rapid descent phase (see above), the mean of the time-averaged angle between the defending bird trajectory and the feeder was 6.3 ± 4.8 degrees among trajectories while the mean of the angle between the defending trajectory and the current position of the chased bird was 3.1 ± 2.3 degrees. The mean of the difference between these angles was 3.2 ± 4.1 degrees, significantly different from 0 (t-test; p = 0.0001). The significance of this result was not sensitive to the elevation threshold used to identify the initial rapid descent phase.

## Discussion

On the whole, our results from the free-flight trajectory kinematics of Ruby-throated Hummingbirds support hypotheses of maximum speed, flight force, acceleration and kinematic power derived from prior laboratory studies. In two cases, maximum flight force and kinematic power, the trajectories reveal that hummingbirds reach their laboratory maxima during typical field behavior, suggesting that these are more relevant performance measures for natural behavior than maximum level flight speed, which was not reached. We also found that hummingbirds vary how they partition gains in speed and elevation in chased versus freely-departing flights but do not vary their overall kinematic power output. This further supports power as a limiting factor in these interactions, as is suggested by a broader assessment of competitive success in a range of hummingbird species [26].

### Kinematics of chased versus non-chased departures

We hypothesized that bird trajectories departing from the feeder while being chased should exhibit greater accelerations, flight speeds, and changes in kinetic and potential energy than those departing alone because high flight speeds and accelerations are aerodynamically costly, generating additional drag or elevating induced power requirements, and offer little apparent benefit for freely-departing birds. The first two of these hypotheses were supported by the data while the third, greater kinematic power expenditure, was not.

Despite differences in speed and acceleration (Figs 3, 5 and Table 1), chased birds and freely-departing bird trajectories both used approximately the same amount of energy during departure (Fig 7A), but partitioned kinetic and potential components differently (Fig 7B and 7C). The chased trajectories appear to maximize kinetic energy over potential energy, while freely-departing trajectories balance both. Thus, the chased birds appear to exhibit a strategy for rapidly achieving an escape speed without increasing their overall energy expenditure compared to a free-departure trajectory.

### Kinetic power output and flight force

Our measures of kinematic power output further support the energetic equivalence of freely-departing and chased bird trajectories, with both classes having statistically identical average kinematic power output. Our whole-bird mass-specific power results also provide some additional data for comparison to other measures of power output based on metabolic and aerodynamic measurements from other studies. These studies quantify many costs beyond the resultant change in kinematic and potential energy state measured here such as the cost of producing lift, cost of drag and efficiency of the flight muscle. Thus, our whole-body power measurements should be much less than these other common measures of muscle or aerodynamic power. However, our kinematic power measures can help delineate what fraction of metabolic power expenditure could be due to other factors. For example, the whole-body metabolic power requirements of Ruby-throated Hummingbirds range from ~258 to 322 W kg^-1^ when flying in variable density gas mixtures [27] whereas the aerodynamic power requirement for hovering flight in the same species was reported as 55 W kg^-1^ in a recent computational fluid dynamics (CFD) study [28]. Our recordings revealed an average kinematic power output of ~19 W kg^-1^ for birds departing from the feeder location with a maximum instantaneous power of ~40 W kg^-1^, similar to the ~44 W kg^-1^ kinematic power for reported for male Anna’s hummingbirds engaged in courtship displays [29]. When compared with the metabolic and aerodynamic measurements noted above, these results support our expectation that kinematic power observations should be much less than metabolic power. However, the difference between our average and peak kinematic power measurements is ~20 W kg^-1^, similar to the difference between peak and hovering metabolic power measurements given the 20% muscle efficiency implied by the CFD result in conjunction with hovering metabolic data. Thus, hummingbirds appear to regularly burst to near their maximum sustainable power during typical flight behavior.

Kinematic power was quantified for many bird species during vertical escape flights following capture [30]; the mean hummingbird feeder-departure power of ~19 W kg^-1^ is similar to that extrapolated from the linear regression of all vertical escape species mean responses. However, the maximum reported hummingbird value is substantially less than the extrapolated maximum vertical escape value; this might reflect the absence of anaerobic muscle in hummingbirds or differences in kinematic data analysis methods.

A second case where laboratory measurements of flight performance can be compared to our free-flight data lies in flight force as quantified by load lifting tests in lab and acceleration in the field. Hovering Ruby-throated Hummingbirds can support an additional load of approximately one body weight [31], implying a flight force surplus sufficient to accelerate an unloaded bird at ~10 m s^-2^ horizontally or ~20 m s^-2^ if foregoing weight support. Furthermore, wild-caught Red-billed Streamertail hummingbirds achieved linear accelerations of up to ~20 m s^-2^ during startle escapes from a feeder [32]. In contrast, the freely-departing trajectories in our field recordings reveal a rather constant linear acceleration of ~8 m s^-2^, beginning when flight speed is still less than 1 m s^-1^. Chased birds achieved similar accelerations at this speed, but also continued to increase speed while losing elevation. These results suggest that the hummingbirds used most (~80%) of their available maximum flight force when departing from the feeder, even when not chased but did not forego weight support.

Centripetal forces also provide another measure of flight force, but since maneuvering forces were greatest at faster flight speeds in the bird trajectories recorded here and aerodynamic forces theoretically scale proportional to the square of velocity, they cannot be compared to hovering load tests or initial departure accelerations. However, the maximum centripetal forces recorded here of 3.9 body weights is substantially less than the maximum reported in recordings of free flight in Cliff Swallows (7.8 body weights) [13] and also from the courtship dive maneuvers of Anna’s Hummingbirds (~9 body weights)[12]. Thus, the competitive intraspecific interactions recorded here from Ruby-throated Hummingbirds may not be a good model for studying maximum forward flight maneuvering performance.

### Maximum speeds

Our hypothesis that the highest flight speeds would be found in the bird defending the feeder was supported. The average maximum speed for a defending bird was found to be 9.2 ± 1.6 m s^-1^, whereas the average maximum speeds for non-chased birds and chased birds were 5.4 ± 1.4 m s^-1^ and 6.2 ± 1.3 m s^-1^, respectively. The maximum speed recorded for a defending bird was 12.0 m s^-1^. This is less than the maximum speed of ~15 m s-1 reached by this species in wind tunnel flights [33] and much less than the greater than 20 m s^-1^ speeds achieved by diving Anna’s Hummingbirds during mating displays [12], a more comparable case since the defending and displaying birds both take advantage of gravitational potential energy to power their flights. The mean maximum speed reached by freely-departing birds here (5.4 m s^-1^) was well within the flight range exhibited by Rufous Hummingbirds flying in a wind tunnel over a range of speeds [34], but not at any well-defined minimum flapping frequency or minimum flapping amplitude within the speed range.

### Guidance and targeting

Our initial hypothesis, based on preliminary observation of hummingbird interactions and the expected priority of defending the feeder over targeting the encroaching bird, was that the defending bird would initially fly toward the feeder, not the encroaching and subsequently chased bird, was not supported. Although the chased bird and feeder were in close proximity for much of the initial descent phase, the flight trajectory of the defending bird was significantly closer to the position of the chased bird than the feeder. We rarely recorded a maneuver by the defending bird to turn and follow the departing chased bird; most defending bird trajectories ended with the bird returning to a level or slightly upward trajectory in approximately the same horizontal direction as its initial descent.

### Differences among sexes

Ruby-throated Hummingbirds are sexually dimorphic, with males having smaller body size, more pointed wings and higher wingbeat frequencies. These differences are associated with differences in flight performance in laboratory environments [35] which might also exist in field flight behavior. However, the typical behavioral roles adopted by the birds in a natural environment make comparisons difficult since most defending birds are male as are most freely-departing birds while most chased birds are female, potentially confounding the comparisons of chased and freely departing birds if females prefer a descending departure trajectory while males prefer an ascending one. The only case where a sufficient sample of both sexes was collected to allow a statistical comparison was for chased birds, 28 of which were female while 5 were male. In this case, peak accelerations occurred at similar times with males exhibiting a non-significantly greater maximum acceleration (15.6 ± 4.1 *vs*. 14.4 ± 4.1 m s^-2^, p = 0.6607) and average kinematic power (23.3 ± 4.0 *vs*. 19.7 ± 9.0 W kg^-1^, p = 0.3988). Differences in average kinematic power during departure among chased and freely departing males approached significance for the previously defined departure duration (23.3 ± 4.0 *vs*. 18.1 ± 5.0 W kg^-1^, p = 0.0619), although not for slightly longer durations.

### Limitations of the study

Interpretation of the results of this study faces two primary limitations—use of data from only one recording location and uncertainty regarding the behavioral motivations of the birds and how these may vary by the sexes of the birds involved in a particular encounter. Use of only one site limits the generality of some aspects of the results; for example a different study site with a different set of perches available for a defending bird would produce different flight trajectories and possibly energy profiles given a difference in potential energy available to a defender. Furthermore, use of only one site restricts the number of birds studied and our data set certainly contains repeated measurements of the same bird. Since the birds themselves were not marked in an individually identifiable manner, we are not able to statistically correct for this effect, so some of our findings may reflect idiosyncratic differences among individuals rather than differences associated with the environment or behavior. For example, all recordings of a defender coming from the typical perch could be a single bird with a greater flight speed than other, casual, defenders who used other starting points.

We also do not know the precise behavioral motivation underlying the different bird-bird interactions we recorded. We analyzed our results in a resource competition framework where birds are defending or encroaching upon the feeder, emphasizing the territorial aspects of hummingbird behavior. However, it is also plausible that the typically male “defender” is in fact performing a limited mating display to the typically female “encroaching” bird. Ruby-throated hummingbird mating displays do include a “U-shaped” plummet and climb [22] by the male near a perched female; this behavior was observed only once at the site during a scouting trip prior to the recording days but does bear some resemblance to the plummet and swoop defending bird trajectory described here. Mating displays in the related Anna’s Hummingbird (*Calypte anna*) do include a dive followed by a chase [36], again somewhat similar to what was recorded here although we did not observe extended chases of females by males. We also found no statistical evidence for differences in flight kinematics based on the sex of the birds involved, but as noted above our statistical power to test for these effects was quite limited.

However, our overall findings that freely behaving hummingbirds—whatever their motivation—use flight speeds much less the maximum achieved in laboratory wind tunnels close to their expected maximum power output and act to use stored potential energy to enhance the power available for types of maneuver. Examination of the effects of local environment and differences in performance among known individuals form interesting topics for further study given the results presented here.

## Supporting Information

S1 Dataset Field Flight Dynamics of Hummingbirds dataset. This archive contains the 3D trajectory data files used as the basis for the analysis described in the manuscript. The data are provided in a directory hierarchy where each recording day has a separate directory and each recording within the day a separate subdirectory. These subdirectories contain a v7 MATLAB data file "dataOut.mat" with a single struct variable "data" containing fields with the camera recording frequency, the raw xyz coordinates, filtered xyz coordinates, velocities and accelerations. Note that GNU Octave and Python can read v7 MATLAB data files. Also provided are files specifying the trials used for different comparisons and the 3D position of the feeder in each recording.
(ZIP)
