# Supplementary material for: Field Flight Dynamics of Hummingbirds during Territory Encroachment and Defense
Source: PLoS One. 2015 Jun 3;10(6):e0125659. doi: 10.1371/journal.pone.0125659 (PMC4454523; doi:10.1371/journal.pone.0125659)
Supplement: S1 Dataset — This archive contains the 3D trajectory data files used as the basis for the analysis described in the manuscript. The data are provided in a directory hierarchy where each recording day has a separate directory and each recording within the day a separate subdirectory. These subdirectories contain a v7 MATLAB data file "dataOut.mat" with a single struct variable "data" containing fields with the camera recording frequency, the raw xyz coordinates, filtered xyz coordinates, velocities and accelerations. Note that GNU Octave and Python can read v7 MATLAB data files. Also provided are files specifying the trials used for different comparisons and the 3D position of the feeder in each recording. (ZIP) [file pone.0125659.s001.zip › dataset/README.rtf]

README file for the dataset archive for "Field Flight Dynamics of Hummingbirds During Territory Encroachment and Defense" submitted to PLoS ONE for consideration of publication.
Description: This archive contains the 3D trajectory data files used as the basis for the analysis described in the manuscript to which it is attached as supplementary material.
License: PLoS content, including this supplementary material, is licensed as "CC-BY"; consult the journal licensing description for further information.
Data structure: The data are provided in a directory hierarchy where each recording day has a separate directory and each recording within the day a separate subdirectory. These subdirectories contain a v7 MATLAB data file "dataOut.mat" with a single struct variable "data" containing fields with the camera recording frequency, the raw xyz coordinates, filtered xyz coordinates, velocities and accelerations. Note that GNU Octave can read v7 MATLAB data files.  Also provided are the following files:
datafile_index.csv - A numbered list of all the datafiles in the hierarchy, numbers are referred to in the following lists of trials with specific behaviors, etc.
all_competitive_trials.csv - A list of all the 2-bird competitive trials
defending_bird_fromperch.csv - A list of all the trials where the defending bird originated at the typical perch
defending_bird_notperch.csv - Trials where the defending bird did not originate at the typical perch
female_chased.csv - Trials where the chased bird was female
female_freely_departing.csv - Trials where the freely departing bird is female
freely_departing.csv - Trials with a free departure behavior
male_chased.csv - Trials where the chased bird is male
male_freely_departing.csv - Trials where the freely departing bird is male
unknownsex_freely_departing.csv - Trials where the sex of the freely departing bird could not be determined from the video
feederPositions.txt - The X-Y-Z coordinate of the base of the feeder for each recording day
